# Liver Injury Patterns and Hepatic Toxicity among People Living with and without HIV and Attending Care in Urban Uganda

**DOI:** 10.1155/2023/6717854

**Published:** 2023-01-28

**Authors:** Clara Wekesa, Rosalind Parkes-Ratanshi, Gregory D. Kirk, Ponsiano Ocama

**Affiliations:** ^1^Infectious Diseases Institute, Makerere University, Kampala, Uganda; ^2^Cambridge University, Institute of Public Health, Cambridge, UK; ^3^John Hopkins University, Baltimore, USA; ^4^College of Health Sciences, Makerere University, Kampala, Uganda

## Abstract

**Introduction:**

The evaluation of the patterns of liver injury, derived from liver chemistry panels, often may narrow on probable causes of the liver insult especially when coupled with clinical history, examination, and other diagnostic tests.

**Methods:**

Among people living with and without HIV and attending care, we used the *R* ratio to evaluate for liver injury patterns. Liver injury patterns were defined as cholestatic (*R* < 2), mixed (*R* = 2‐5), and hepatocellular (*R* > 5).

**Results:**

Overall, the proportions of participants with cholestatic liver injury, mixed liver injury, and hepatocellular liver injury were 55%, 34%, and 4%, respectively, with similar distribution when stratified by HIV status. Alcohol use among participants without HIV was associated with all patterns of liver injury (cholestatic liver injury (OR = 4.9 CI (1.0-24.2); *p* = 0.054), mixed liver injury (OR = 5.3 CI (1.1-27.3); *p* = 0.043), and hepatocellular liver injury (OR = 13.2 CI (1.0-167.3); *p* = 0.046)). Increasing age was associated with cholestatic liver injury among participants with HIV (OR = 2.3 CI (1.0-5.3); *p* = 0.038). Despite a high hepatitis B prevalence among participants with HIV, there was no association with liver injury.

**Conclusions:**

Liver injury is prevalent among both people living with and without HIV in care, and cholestatic liver injury is the most common pattern. Alcohol is associated with all patterns of liver injury and increasing age associated with cholestatic liver injury among people living without HIV and people living with HIV, respectively.

## 1. Introduction

Liver disease is of growing concern globally and it would appear that people living with HIV (PLHIV) are indiscriminately affected, having a higher prevalence and a higher risk for acceleration to liver disease complications [1]. Liver biopsy remains the gold standard for the evaluation of liver pathology; however, its use is limited in resource constrained settings due to expense incurred in its conduct and the lack of expertise in conducting the procedure. In addition, liver biopsy is an invasive procedure that is not readily accepted by patients, and it carries the risk of bleeding and is prone to sampling errors [2]. Recent advancements are geared towards noninvasive techniques of evaluation of liver disease as they are readily acceptable and can be done repetitively to assess disease progression. Liver chemistry panel is readily accessible even within resource-constrained settings and is often embedded in routine care of patients suspected of having liver disease. It is common for ambulatory patients to have elevated liver enzymes without obvious signs of liver disease especially in the context of HIV/AIDS [3, 4]. Evaluation for liver injury patterns based on liver chemistry parameters provides guidance on approach of diagnostic efforts geared towards narrowing down on possible causes of the observed liver injury [5].

Commonly described patterns of liver injury include hepatocellular, cholestatic (or biliary), and hepatobiliary (mixed), the descriptions of which are derived from computations of the parameters of a liver chemistry panel. Pattern descriptions are based on which liver enzymes are predominantly elevated in comparison to others. For example, hepatocellular liver injury is mainly characterized by significant elevation of the liver transaminases (ALT, AST), occurring as an isolated event or higher than the other liver chemistry parameters. This type of liver injury signifies damage to the hepatocytes. In cholestatic liver injury, we mainly observe an elevation of the alkaline phosphatase serum levels (ALP) and gamma glutamyl transferase (GGT) which involves mainly the bile duct or presents as infiltrative disease [5, 6]. A number of liver diseases can present with the same pattern of liver injury; thus, a detailed clinical history, examination, and other diagnostic procedures remain necessary for a more comprehensive assessment in narrowing down on a possible cause [5]. Viral hepatitis, drug-induced hepatitis, alcoholic liver disease, and nonalcoholic hepatosteatosis usually present as hepatocellular liver injury. Cholestatic liver injury is mainly caused by disease obstructing the biliary system like primary biliary cirrhosis or sclerosing cholangitis, gallstones, autoimmune hepatitis mainly involving the biliary system, infiltrative diseases like sarcoidosis, granulomatous infections like tuberculosis, and cancers like Hodgkin's lymphoma. Drug-induced liver injury from certain drugs may also at times present as cholestatic liver injury [6].

Some studies conducted during the time of limited ART access found that cholestatic liver injury was the most common form of liver injury among HIV-infected patients in care. This was mainly due to opportunistic infections and carcinomas such as TB coinfection and hepatocellular carcinoma as well as from ART drug toxicities [1]. In the recent years, there has been a scale up on ART coverage, improved drug formulations with less toxicity profiles including to the liver, early ART initiation at higher CD4 counts, and use of ART drug combinations that have duo activity against HIV and HBV. These developments have played a key role in halting progression of chronic hepatitis B infection, lowering the incidence of drug-induced liver injury, and maintaining relatively good immune function against opportunistic infections and malignancies. In light of these positive developments that may reduce incidence of liver injury, there is adoption of other characteristics that still pose a risk to liver health. These include the use of alternate medicines, such as herbal therapies, and some of which may be toxic to the liver and others of whose effects are largely unknown and may possibly be antifibrotic in nature, increasing over-the-counter prescriptions and polypharmacy with limited control on appropriate use and emerging lifestyle risk factors such as obesity that predispose to fatty liver disease. It is important to note that the distribution of these non-HIV risk factors is similarly common within the general population.

Placing these non-HIV risk factors into consideration, it may be possible that the patterns of liver injury, especially among PLHIV, have since changed, and an evaluation of current patterns may give insight to possible predominant causes of liver disease presently. We conducted a cross-sectional study among people living with and without HIV to determine the burden of the different patterns of liver injury, their associated factors, and the burden of hepatic toxicity as assessed using the AIDS Clinical Trials Group (ACTG) classification.

## 2. Methods

### 2.1. Study Design and Setting

We conducted a cross-sectional study between January 2015 and March 2020 involving PLHIV and people without HIV and attending outpatient clinics within the National Referral Hospital in Kampala, Uganda. The PLHIV were enrolled from a tertiary HIV care clinic within Mulago National Referral Hospital that has been in existence for the last 14 years and has over 95% ART coverage. The people living without HIV were enrolled from the Ear Nose and Throat (ENT) outpatient clinic at Mulago National Referral Hospital that receives mainly self-referrals from the surrounding area as well as consultations.

### 2.2. Study Population

We enrolled a total sample size of 516 participants, 260 PLHIV and 256 people living without HIV matched on age group and gender. The participants with HIV were sampled from an existing database for an ongoing prospective study on progression of premalignant cirrhosis. Using the age and gender demographic of the participants with HIV, we then consequently enrolled the participants without HIV matched on these demographics. Eligible participants had to be 18 years and above with no prior history of liver disease. The enrolment of participants with HIV was conducted within an adult care clinic, and matching for the participants without HIV had to be similar to provide suitable comparison. Pregnant women and participants with medical implants were excluded from participation as the use of the FibroScan is not recommended in these patient groups.

### 2.3. Study Procedures

The data collection and sample processing were similar for both participants with and without HIV. Information for the participants with HIV was collected retrospectively from an existing database and blood samples from a repository used for testing. The participants without HIV were actively enrolled, and similar information and blood samples were collected at the time of enrollment.

#### 2.3.1. Sociodemographic and Clinical Information

A standardized questionnaire was used to collect the following information: sociodemography (age and gender); lifestyle habits (self-reported use of alcohol, tobacco, and herbal medicines); and ART use, results on viral hepatitis (participants with HIV), and anthropometric measurements (weight and height).

#### 2.3.2. Blood Sampling

Liver biochemistry was analyzed using Hitachi Cobas C311. Hepatitis B serology was performed using an enzyme immunoassay (Monolisa HBsAg Ultra 3; Bio-Rad). Hepatitis C antibody testing was done using 3rd generation enzyme immunoassay (Bio-Rad Monolisa Anti-HCV PLUS). A confirmatory HIV serology test was performed for the HIV-uninfected participants.

#### 2.3.3. Categorizing Liver Injury Patterns

Using parameters from the liver chemistry alanine transaminase (ALT) and alkaline phosphatase (ALP), we computed an *R* ratio using the formula below following American College of Gastroenterology Clinical guidelines [7].

(1) $$\begin{array}{c} R=\frac{\text{ALT}/\text{ALT ULN}}{\text{ALP}/\text{ALP ULN}}, \end{array}$$

where ULN is the upper limit of normal as provided by the laboratory.

Liver injury patterns were defined as cholestatic (*R* < 2), mixed (*R* = 2‐5), and hepatocellular (*R* > 5).

To evaluate for hepatic toxicity, we applied the AIDS Clinical Trials Group (ACTG) classification for hepatic toxicity as elaborated below (Table 1) [4].

### 2.4. Analysis

Description of continuous data was presented as means and median and categorical data as percentages. Between-group comparisons were done using chi square testing and significance set as *p* < 0.05. Liver injury patterns were presented as proportions and comparisons between groups made using chi square testing. Data on hepatotoxicity and cholestasis were also presented as percentages and between-group comparisons done using chi square testing.

To determine what variables predicted the presence of the various categories of liver injury patterns (cholestatic, mixed, and hepatic liver injury), we fitted a multinomial logistic regression model since we had more than two outcomes. We used the category of participants without liver injury as the reference group for which all comparisons were made. Analyses were also stratified by HIV status. To determine which variables where to be used in the final models in each strata, we deployed forward elimination process using likelihood ratio testing for each variable added into the model and set significance level at 0.05. If the level of significance in the testing of the model with and without the variable was ≤0.05, the variable was retained in the final model, and if greater than 0.05, it was dropped. Gender and age were retained in the models regardless of whether the significance of the likelihood ratio testing was greater than 0.05.

## 3. Results

There was equal gender distribution between the two study populations, and the overall mean age was 44 years (SD ± 10.3). The proportion of persons who had ever consumed alcohol and used tobacco products was similar regardless of HIV status. The participants without HIV had higher mean BMI (27 vs. 23; *p* < 0.001), more use of herbal medicines in the last 12 months (33% vs. 25%; *p* = 0.04), and higher median serum cholesterol levels (total cholesterol 176 mg/dL vs. 162 mg/dL; *p* < 0.001; LDL cholesterol 102 mg/dL vs. 96 mg/dL; *p* = 0.022). Participants with HIV had over four times higher prevalence of chronic hepatitis B infection (14% vs. 3%; *p* < 0.001) and higher median serum levels of liver enzymes (ALT 20 U/L vs. 17 U/L, *p* < 0.001; ALP 90 U/L vs. 77 U/L, *p* = 0.004) (Table 2).

Participants with HIV were more likely to have hepatotoxicity compared to participants without HIV, and this difference was of borderline significance. There were no differences in the distribution of participant proportions for the different grades of cholestasis. None of the participants had life-threatening hepatoxicity (AST > 10 times ULN), but 0.4% (2/256) of the participants with HIV presented with life-threatening cholestasis (serum bilirubin > 5 times ULN) (Table 3).

**Figure 1:**
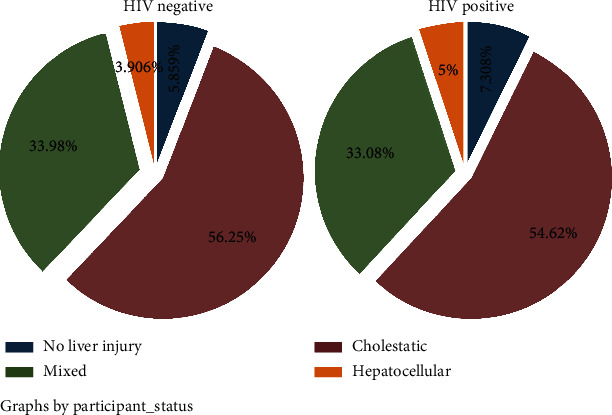
Distribution of liver injury patterns among adults with and without HIV attending outpatient care at Mulago Hospital (2015-2020).

Overall, the proportions of participants with cholestatic liver injury, mixed liver injury, and hepatocellular liver injury were 55%, 34%, and 4%, respectively. The pattern of liver injury was similar when stratified by HIV status (Figure 1). Female participants had a significantly higher proportion of cholestatic liver injury (61% vs. 48%; *p* = 0.023), and male participants had a significantly higher proportion of mixed liver injury (39% vs. 29%; *p* = 0.023).

Among participants without HIV, the use of alcohol (previous and current) was a predictor for all patterns of liver injury (cholestatic, mixed, and hepatocellular). Alcohol use among participants without HIV was associated with 5 times the odds of having cholestatic liver injury (OR = 4.9 CI (1.0-24.2); *p* = 0.054), 5 times the odds of mixed liver injury (OR = 5.3 CI (1.1-27.3); *p* = 0.043), and 13 times the odds of hepatocellular liver injury (OR = 13.2 CI (1.0-167.3); *p* = 0.046) compared to participants with HIV and no liver injury. There was borderline significance of CAP score ≥ 248 dB/m (>11% of fat infiltration in the liver) being a predictor of cholestatic liver injury (OR = 1.0 CI (0.9-1.0); *p* = 0.067) among participants without HIV compared to participants without HIV and liver injury. Among participants with HIV increasing age had 2 times the odds of having cholestatic liver injury (OR = 2.3 CI (1.0-5.3); *p* = 0.038) compared to participants with HIV but with no liver injury (Table 4).

## 4. Discussion

We found that the most predominant pattern of liver injury was cholestatic liver injury even when the population was stratified by HIV status (50% among HIV-infected and 53% among HIV-uninfected). This distribution of liver injury pattern has been similarly demonstrated in earlier studies conducted among PLHIV in care in Uganda and Ethiopia [1, 8]. The least common pattern of liver injury was hepatocellular injury. In contrast, studies conducted in Nepal among PLHIV focused on drug-induced liver injury and demonstrated hepatocellular liver injury pattern to be the most predominant pattern of liver injury [3]. Study participants enrolled in the former study were ART-naïve patients who were followed up for a period of time. It is possible that with ART initiation, there could have been increased risk of immune reconstitution syndrome involving the liver tissue and/or increase risk of hepatotoxicity from certain ART drug classes that often occurs at initiation.

Predictors of cholestatic liver injury among participants with HIV were found to be increasing age. In earlier studies in Uganda, the associated factors for the observed cholestatic liver injury included disseminated tuberculosis, viral hepatitis B and C, and adverse drug reactions, particularly from nevirapine and isoniazid. We attribute these differences to the timing of our study, being conducted at a time of improved ART coverage with early initiation of ART reducing risk for opportunistic infections and malignancies, change in ART formulations with less liver toxicity, and satisfactory action against viral hepatitis B infection including routine testing of HBV [1]. Studies from Ethiopia evaluating drug-induced liver injury demonstrated ART use to be associated with cholestatic liver injury among PLHIV and hepatocellular liver injury patterns among PLHIV coinfected with TB [8]. In our study, the use of ART and co-trimoxazole was not predictive of liver injury. It is possible that there were differences in ART regimen and hence safety profile. In addition, majority of the participants in the Ethiopian study had CD4 counts below 200 cells/mm^3^, a risk factor for immune reconstitution syndromes including unmasking tuberculosis and other infections affecting liver chemistry. Hepatitis B infection is a predisposing risk factor for drug-induced liver injury; however in our study, it was not associated with any of the patterns of liver injury among the PLHIV in whom it was most prevalent. Given that the current ART regimen contains 2 drugs with activity against HBV infection and reduced risk of resistance, it is possible that the participants with HIV and coinfected with HBV had controlled or suppressed infection and hence lowered risk for drug-induced liver injury from ART and concomitant drugs [9, 10]. Among the participants without HIV, the use of alcohol was associated with all patterns of liver injury. The presence of more than 11% fat content in the liver (CAP score ≥ 248 dB/m) had a marginal association with the presence of cholestatic liver injury. High CAP scores are indicative of NAFLD which from other studies is an independent risk factor for cholestatic liver injury and a risk factor for drug-induced liver injury [9].

The proportion of participants with HIV having hepatotoxicity was slightly higher than that of participants without HIV. This could be attributed to the fact that PLHIV usually have higher serum transaminase levels [11, 12]. The majority of participants had mild and moderate hepatotoxicity, and none of the participants had life-threatening hepatotoxicity. This finding is similar to that found in other observational studies among patients with HIV that showed predominantly grades 1 and 2 hepatotoxicity [4, 12]. The overall proportion of hepatotoxicity among patients with HIV was 14% which was lower than found in other studies [12].

The limitations in our study included, not being able to quantify the duration, amounts of alcohol consumed and coadministered substances such as herbal medicines with alcohol as this would have provide better indicative of the role of alcohol in causation of liver injury. We were unable to measure CAP scores among the participants with HIV given the difference in timing of data collection, apart from herbal medicines, not evaluating for concomitant drug use among the participants without HIV and the limited capacity to perform liver biopsies for pathological review due to lack of financial support. We also did not test for schistosomiasis a common infection in this setting. The study however does provide information on liver injury patterns in a more current time of improved ART coverage and influence of non-HIV risk factors.

### 4.1. Key Finding

Cholestatic liver injury is the predominant pattern of liver injury among PLHIV and people without HIV in care. The use of alcohol seems to be associated with the whole spectrum of liver injury among people living without HIV. Despite a high prevalence of HBV infection, it was not associated with liver injury among PLHIV. Slightly elevated serum transaminases are a common occurrence especially among PLHIV.

We recommend the creation of awareness programs that educate on the effects of non-HIV risk factors such as alcohol use on liver health, especially among people living without HIV. We recommend that even with future transitions of ART regimen that the use of ART drugs with duo effect on HBV infection be maintained. We also recommend the conduct of further studies that make use of pathological evaluation (using liver biopsy as gold standard) to adequately define aetiology of liver injury patterns observed.

## Figures and Tables

**Table 1:** AIDS Clinical Trials Group (ACTG) classification of hepatotoxicity and cholestasis.

Hepatotoxicity	Serum ALT	Cholestasis	Serum bilirubin
Grade 0	1.25 × ULN	Grade 0	<1.1 × ULN
Grade 1	1.25 − 2.5 × ULN	Grade 1	1.1 − 1.5 × ULN
Grade 2	2.6 − 5 × ULN	Grade 2	1.6 − 2.9 × ULN
Grade 3	5.1 − 10 × ULN	Grade 3	3 − 5 × ULN
Grade 4	>10 × ULN	Grade 4	>5 × ULN

ALT: alanine transferase; ULN: upper limit normal. Grade 4 Life threatening Grade 1 Mild; Grade 2 Moderate; Grade 3 Severe.

**Table 2:** Baseline social demographic and clinical characteristics of adults with and without HIV and in care at Mulago Hospital Uganda (January 2015-March 2020).

Characteristic	Participants without HIV *n* (%)	Participants with HIV *n* (%)	Overall *N* (%)	*p* value
	256 (49.6)	260 (50.4)	516 (100)	
Gender				
Female	142 (55)	145 (56)	287 (56)	0.945
Male	114 (45)	115 (44)	229 (44)	
Mean age (years SD±)	44 (10.2)	44 (10.3)	44 (10.3)	0.577
Mean BMI (years SD±)	27 (10.6)	23 (4.5)	25 (8.2)	**<0.001**
Ever consumed alcohol	112 (44)	106 (41)	218 (42)	0.493
Ever used tobacco products	44 (17)	40 (15)	84 (16)	0.579
Used herbal medicines in the last 12 months	85 (33)	65 (25)	150 (29)	**0.040**
HBsAg Positive	7 (3)	36 (14)	43 (8)	**<0.001**
HCV antibody Positive	8 (3)	7 (3)	15 (3)	0.770
Median ALT (IQR)	17 (13-24)	20 (14-26)	18 (14-26)	**<0.001**
Median ALP (IQR)	77 (63-99)	90 (66-114)	82 (64-106)	**0.004**
Median GGT (IQR)				
Median TC (IQR)	176 (159-200)	162 (132-190)	173 (147-196)	**<0.001**
Median LDL-C (IQR)	102 (87-122)	96 (70-112)	98 (77-120)	**0.022**
Median TG (IQR)	118 (85-164)	119 (83-160)	118 (84-161)	**<0.001**
Median HDL-C (IQR)	47 (40-56)	40 (31-48)	44 (35-53)	**0.010**

BMI: body mass index; HBsAg: hepatitis B surface antigen; HCV: hepatitis C; ALT: alanine transferase; ALP: alkaline phosphatase; GGT: gamma glutamyl transferase; TC: total cholesterol; LDL-C: low-density lipoprotein cholesterol; TG: triglycerides; HDL-C: high-density lipoprotein cholesterol.

**Table 3:** Distribution of hepatic toxicity and cholestasis among adults with and without HIV attending care at Mulago Hospital Uganda (2015-2020).

	Hepatotoxicity^∗^				Cholestasis^∗^			
	Participants without HIV *n* (%)	Participants with HIV *n* (%)	All *n* (%)	*p* value	Participants without HIV *n* (%)	Participants with HIV *n* (%)	All *n* (%)	*p* value
Grade 0	236 (92)	223 (86)	459 (89)	0.072	237 (94)	221 (95)	458 (95)	0.361
Mild	13 (5)	23 (9)	36 (7)		8 (3)	3 (1)	11 (2)	
Moderate	7 (3)	11 (4)	18 (3)		5 (2)	5 (2)	10 (2)	
Severe	0 (0)	3 (1)	3 (1)		2 (1)	1 (0.4)	3 (0.6)	
Life threatening					0 (0)	2 (1)	2 (0.4)	

^∗^AIDS Clinical Trials Group (ACTG) classification of hepatotoxicity and cholestasis.

**Table 4:** Predictors of liver injury patterns among adults with and without HIV attending outpatient care at Mulago Hospital (2015-2020).

Variable	Cholestatic liver injury			Mixed liver injury			Hepatocellular liver injury		
	OR	95% CI	*p* value	OR	95% CI	*p* value	OR	95% CI	*p* value
	Participants without HIV								
Male gender	0.5	(0.1-1.7)	0.291	1.1	(0.3-4.0)	0.906	0.1	(0.01-1.2)	0.07
Ever used alcohol	4.9	(1.0-24.2)	**0.054**	5.3	(1.1-27.3)	**0.043**	13.2	(1.0-167.3)	**0.046**
Used herbs in the last 12 months	606		0.987	498		0.987	139		0.988
Total cholesterol	1.0	(0.9-1.0)	0.888	1.0	(0.9-1.0)	0.57	1.0	(0.9-1.1)	0.673
LDL-C	1.0	(0.9-1.1)	0.386	1.0	(0.9-1.1)	0.254	1.0	(0.9-1.1)	0.993
CAP score ≥ 248 dB/m	1.0	(0.9-1.0)	0.067	1.0	(0.9-1.0)	0.353	1.0	(0.9-1.0)	0.331
	Participants with HIV								
Male gender	0.6	(0.2-2.3)	0.517	2.0	(0.5-7.4)	0.299	8.7	(0.7-113.3)	0.098
Increasing age	2.3	(1.0-5.3)	**0.038**	1.1	(0.5-2.6)	0.775	1.1	(0.3-4.5)	0.915
cART	0.6	(0.2-2.8)	0.536	0.9	(0.2-4.2)	0.906	1.8	(0.1-25.3)	0.662
Nadir CD4 > 200 cells/mm^3^	0.7	(0.2-2.2)	0.537	1.3	(0.4-4.4)	0.659	0.8	(0.1-6.1)	0.851
Using Septrin prophylaxis	1.90*E* − 06		0.986	1.60*E* − 06		0.986	0.5		1.000

^∗^Outcome treated as multicategory and base group and/or reference group is participants with no liver injury.

## Data Availability

The data used to support the findings of this study are available from the corresponding author upon request.

## Abbreviations

ACTG: AIDS Clinical Trials Group
AIDS: Acquired immunodeficiency syndrome
ALP: Alkaline phosphatase
ALT: Alanine transaminase
ART: Antiretroviral therapy
AST: Aspartate transaminase
BMI: Body mass index
CAP: Controlled attenuated parameter
ENT: Ear nose and throat
GGT: Gamma glutamyl transferase
HBV: Hepatitis B infection
HCV: Hepatitis C infection
HDL: High-density lipoprotein
HIV: Human immunodeficiency virus
LDL: Low-density lipoprotein
PLHIV: People living with HIV
OR: Odds ratio
TB: Tuberculosis
TC: Total cholesterol
TG: Triglycerides
SOMREC: School of Medicine Research and Ethics Committee
ULN: Upper limit normal.
